# Predicting body weight of male Kuroiler chickens from linear body measurements using MARS and CART data-mining algorithms

**DOI:** 10.5194/aab-68-653-2025

**Published:** 2025-10-30

**Authors:** Simushi Liswaniso, Ruth Kasonso, Lubabalo Bila, Madumetja Cyril Mathapo, Oswin Chibinga, Thobela Louis Tyasi, Xue Sun, Rifu Xu, Ning Qin

**Affiliations:** 1 School of Agricultural Sciences, Department of Animal Sciences, University of Zambia, P.O. Box 32379, Lusaka, Zambia; 2 Potchefstroom College of Agriculture, Department of Animal Production, Private Bag X1292, Potchefstroom, 2520, South Africa; 3 School of Agricultural and Environmental Sciences, Department of Agricultural Economics and Animal Production, University of Limpopo, Private Bag X1106, Sovenga 0727, Limpopo, South Africa; 4 Department of Animal Breeding, Genetics and Reproduction, College of Animal Science and Technology, Jilin Agricultural University, Changchun 130118, Jilin, China

## Abstract

Body weight is an essential trait in chickens, especially in markets where chickens are priced based on body weight and where medicine dosages depend on the animal's weight. However, not all farmers can afford scales to measure body weight, and they sometimes lack technical know-how during breakdowns, which is a challenge for small-scale farmers. Lately, data-mining algorithms have been used to help predict live body weight in livestock as they perform better than traditional prediction methods like linear regression. This study, therefore, aimed to develop models to predict the live body weight of the Kuroiler chicken breed from linear body measurements using classification and regression tree (CART) and multivariate adaptive regression spline (MARS) data-mining algorithms and to assess which of the two data-mining algorithms has a superior predictive performance. Linear body measurements were taken using a tailor's tape, and the body weight was taken using an electronic scale from 100 male Kuroiler chickens aged 23 weeks. The linear body measurements taken were corpus length (CL), chest circumference (CC), thigh length (TL), thigh circumference (TC), shank circumference (SC), shank length (SL), keel length (KL), and body length (BL). Results showed that the Kuroiler chickens used had an average live body weight of 2.01 kg, which correlated positively with all measured linear body measurements. Both MARS and CART developed models that included chest circumference, shank length, thigh circumference, and keel length. Furthermore, the MARS and CART models isolated the keel length and chest circumference as the most crucial traits in predicting the body weight of male Kuroiler chickens. The predictive performance showed that CART was the best model, with the highest r , $R^{2}$ , and adjusted $R^{2}$ . These results suggest that the CART data-mining algorithm might help to determine the breeding standards of the Kuroiler chicken breed for a breeding program. The findings of this study may be helpful to breeders, producers, and marketers of Kuroiler chickens.

## 1 Introduction

Indigenous chicken production is an important venture among rural communities, accounting for at least 80 % and 65 % of the poultry production in Africa and Zambia, respectively (Liswaniso et al., 2023). The chickens used are characterized by poor performance, like low egg production, low egg weights, low body weight, and poor growth rate (Matawork, 2018; Muasya et al., 2022; Sebho, 2016; Liswaniso et al., 2020). The poor performance of these chickens has led to the importation of some improved free-range chickens to cross-breed with indigenous chickens. One such breed introduced into Africa in the early 2000s is the Kuroiler breed. One of the earliest places this breed was introduced was East Africa, where it performed very well (Sharma et al., 2015). The Kuroiler breed is a dual-purpose breed initially developed in India. It was purposefully developed for meat and eggs by Kegfarms^®^ Private Ltd (Moto and Rubanza, 2022). Kuroiler chickens, characterized by high body weight, lay their first egg as early as 5 to 6 months and lay up to 200 eggs within 12 to 16 months (Sharma et al., 2015; Bhonsle et al., 2018).

Body weight (BW) remains a trait of economic importance (Yakubu and Ari, 2017) since it translates into cash, especially in markets where chicken prices are on a weight basis. Knowing the body weight also helps in making breeding decisions and also aids in measuring the correct dosages of medications (Norris et al., 2015). However, its measurements may be challenging, especially among small-scale farmers, as it requires weighing scales, which may be challenging for most small-scale farmers to access (Temoso et al., 2017). When found, the need for more technical know-how to maintain them is also a challenge. This calls for scientists to identify ways in which farmers can predict body weight with ease.

Prediction models using linear body measurements have been developed recently to predict body weight (Isaac and Adeolu, 2023; Obike et al., 2019). These used linear regression that depended solely on the association between body weight and linear body measurements (Bila et al., 2023). However, for these traditional ways to be used, there is a need to fulfill some assumptions, such as non-multicollinearity, constant variance, normality, and linearity (Draper and Smith, 1998), which are not always met. Nonetheless, since their advent, data-mining algorithms have become an excellent alternative to regression due to their ability to handle multicollinearity. This is due to their merits in not needing so many assumptions (Mendeş and Akkartal, 2009). Two data-mining algorithms are most extensively employed in animal science. These are the multivariate adaptive regression spline (MARS) and the classification and regression tree (CART).

As a non-parametric regression, MARS was developed by Friedman (1991). It employs the “smoothing spline” technique. This method provides both innovation and substantial ease in interpreting variables and their interactions (Jerome, 1991; Adiguzel and Cengiz, 2023). MARS has been used in livestock sciences to predict the body weight of the Kajli sheep breed (Faraz et al., 2023) and Anatolian buffaloes (Ağyar et al., 2022). The classification and regression tree (CART) algorithm analyzes data for modeling and segmentation purposes. A decision tree is created using rules that group data into unique categories (Ali et al., 2015). CART has been employed in livestock sciences to predict the weight of goats (Oyebanjo et al., 2023; Mathapo et al., 2022) and the egg weight of indigenous chickens (Liswaniso et al., 2021).

Literature on the Kuroiler breed is limited to studies on the production and productivity of these birds. Guni et al. (2021) assessed the performance of Kuroiler chickens under farmer management in Tanzania; Sanka et al. (2021a) studied their egg-laying performance under semi-intensive systems; in another study, Sanka et al. (2021b) studied the meat quality traits of these chickens in semi-scavenging systems; Yakubu and Ari (2017) used principal component and discriminant analyses of body weight and conformation traits; and Kalai et al. (2023) studied the pathomorphology of gonads under chronic glyphosate toxicity. Nonetheless, there is scanty information on the use of MARS and CART in predicting the body weight of Kuroiler chickens despite this being paramount in making more precise decisions regarding the characterization of breeds, genetic resource conservation, and flock management of Kuroiler chickens, harboring prospects for generating sustainable production of meat, especially in the tropics in tropical climates (Faraz et al., 2021). Hence, the objective of this study was to use MARS and CART to predict the body weight of Kuroiler chickens and to compare their predictive performance.

## 2 Materials and methods

### 2.1 Study location

The study was conducted at the University of Zambia village chicken park in the district of Chongwe, about 22 km east of Lusaka, Zambia's capital. Chongwe is a small town dominated by a farming community.

### 2.2 Chickens and their management

Day-old Kuroiler chicks were obtained from a reputable breeder in Zambia and then transported to the University of Zambia's village chicken park. The chicks underwent brooding following all standard practices, including vaccinations against major known diseases. After 10 weeks, chickens were moved into the production unit and reared under a semi-intensive system. Night shelter was provided in a raised housing system. The chickens were allowed to roam freely during the daytime. While the chickens had free unlimited access to water, commercially compounded feed was rationed to the chickens. They did more scavenging for their feed.

### 2.3 Data collection

A sample of 100 male Kuroiler chickens was randomly selected from the flock at 23 weeks, and body weight and some linear body measurement traits were individually taken. Body weights were taken using a weighing scale with an accuracy of 0.01 kg, while linear body measurements were then taken using a tailor's flexible measurement tape. The linear body measurement traits taken were corpus length (CL), chest circumference (CC), thigh length (TL), thigh circumference (TC), shank circumference and keel length (KL), and body length (BL). The body weight and linear body measurement traits were taken according to the FAO (2012) guidelines on the characterization of farm genetic resources, as used in other studies (Semakula et al., 2011).

### 2.4 Statistical analysis

The Statistical Package for Social Sciences (IBM SPSS, 2019) version 26.0 was used to analyze the data for descriptive statistics of the body weight and linear body measurement traits of Kuroiler chickens. The correlation matrix was used to determine the correlation between body weight and linear body.

#### 2.4.1 Multivariate adaptive regression spline (MARS) algorithm

The multivariate adaptive regression spline algorithm was carried out as defined by Bila et al. (2023) and Rashijane et al. (2023). The MARS data-mining algorithm is defined as follows:

1 $$f\left(x\right)=\beta_{0}+\sum_{M=1}^{M}\beta_{m}\prod_{k=1}^{k_{m}}h_{m}(X_{v\left(k,m\right)}),$$

where f(x) is the estimated value of the dependent variable; $\beta_{0}$ and $\beta_{m}$ are the intercept; $h_{m}$ ( $X_{v(k,m)}$ ) is the basis function, where v(k,m) is an index of the predictor for the m th component of the k th product; and K is the parameter regulating the order of interaction. After building the most suitable MARS model, the basic functions that did not contribute much to the model fitting performance were removed in the pruning process based on the following generalized cross-validation error (GCV) (Bila et al., 2023; Eyduran et al., 2019):

2 $$\text{GCV}(\lambda )=\frac{\sum_{i=1}^{n}{\left(y_{i}-y_{ip}\right)}^{2}}{{\left(1-\frac{M\left(\lambda \right)}{n}\right)}^{2}},$$

where n is the number of training cases, $y_{i}$ is the observed value of a response variable, $y_{ip}$ is the estimated value of a response variable, and M(λ) is a penalty function for the complexity of the model with λ terms.

#### 2.4.2 Classification and regression tree (CART) algorithm

As a replicating data-mining algorithm tree, CART was created by repetitively splitting a node into pairs of child nodes, opening with the root node that contains the whole learning sample following Breiman et al. (1984) and Kuhn and Johnson (2013).

#### 2.4.3 Predictive performance of CART and MARS models

The best model between MARS and CART was determined by calculating the goodness-of-fit test criteria (Çelik and Yilmaz, 2018; Bila et al., 2023). The following goodness-of-fit criteria were calculated:

*Pearson's correlation coefficient.*

3 $$r=\frac{\mathrm{cov}(y_{i},y_{ip})}{S_{yi}S_{Yip}}$$

*Relative root mean square error.*

4 $$\mathrm{RRMSE}=\frac{\sqrt{\frac{1}{n}\sum_{i=1}^{n}(y_{i}-y)\times 2}}{\bar{y}}$$

*Mean error.*

5 $$\mathrm{ME}=\frac{1}{n}\sum_{i=1}^{n}(y_{i}-y_{ip})$$

*Performance index.*

6 $$\mathrm{PI}=\frac{\mathrm{rRMSE}}{1+r}$$

*Coefficient of determination.*

7 $$\mathrm{Rsq}=1-\frac{\sum_{i=1}^{n}(y_{i}-{\hat{y}}_{i}{)}^{2}}{\sum_{i=1}^{n}(y_{i}-\bar{y}{)}^{2}}$$

*Adjusted coefficient of determination.*

8 $$\mathrm{ARseq}=1-\frac{\frac{1}{n-k-1}\sum_{i=1}^{n}{\left(y_{i}-{\hat{y}}_{i}\right)}^{2}}{\frac{1}{n-1}\sum_{i=1}^{n}{\left(y_{i}-\bar{y}\right)}^{2}}$$

*Root-mean-square error.*

9 $$\mathrm{RMSE}=\sqrt{\frac{1}{n}\sum_{i=1}^{n}(y_{i}-{\hat{y}}_{i}{)}^{2}}$$

*Standard deviation ratio.*

10 $$\mathrm{SDR}=\sqrt{\frac{\frac{1}{n-1}\sum_{i=1}^{n}(\varepsilon_{i}-\bar{\varepsilon }{)}^{2}}{\frac{1}{n-1}\sum_{i=1}^{n}(y_{i}-\bar{y}{)}^{2}}}$$

*Akaike information criteria.*

11 $$\text{AIC}=N\text{Ln}\left(\frac{\text{SSE}}{N}\right)+2p$$

*Mean absolute percentage error.*

12 $$\text{MAPE}=\frac{1}{n}\sum_{i=1}^{n}\left|\frac{Yi-\hat{Y}i}{Yi}\right|\times 100$$

*Mean absolute deviation.*

13 $$\text{MAD}=\frac{1}{n}\sum_{i=1}^{n}\left|\frac{Yi-\hat{Y}i}{Yi}\right|$$

*Global relative approximation error.*

14 $$\text{RAE}=\sqrt{\frac{\sum_{i=1}^{n}(Yi-\hat{Y}i)2}{\sum_{i=1}^{n}Y\frac{2}{i}}}$$

*Coefficient of variance.*

15 $$\text{CV}=\sqrt{\frac{\frac{1}{n-1}\sum_{i=1}^{n}(\varepsilon_{i}-\bar{\varepsilon }{)}^{2}}{\bar{Y}}}\times 100$$

## 3 Results

Descriptive statistics of the chickens' body weights and linear body measurements are presented in Table 1. The results indicated that the average live body weight of the Kuroiler chicken breed was 2.01 kg.

**Table 1:** Descriptive statistics of body weight and linear body measurements of chickens.

Traits	Mean ± SD	Minimum	Maximum
BW (kg)	2.01±0.32	1.42	2.80
CL (cm)	23.38±1.05	20.80	25.80
CC (cm)	32.25±1.83	28.00	36.30
TL (cm)	16.42±1.02	14.00	19.00
TC (cm)	11.06±1.19	8.70	13.90
SC (cm)	4.66±4.66	4.10	5.10
SL (cm)	12.69±0.69	10.30	14.10
KL (cm)	13.04±0.94	11.00	15.00
BL (cm)	47.24±1.94	42.00	51.10

BW: body weight, CL: corpus length, CC: chest circumference, TL: thigh length, TC: thigh circumference, SC: shank circumference, SL: shank length: KL: keel length:, and BL: body length.

**Figure 1:**
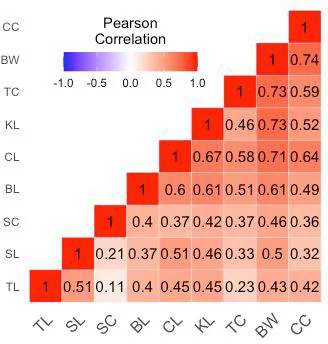
Heat map of body weight correlation of measured traits in chickens. The correlation color demonstration is as follows: high correlation is red, moderate correlation is white, and low correlation is blue. BW: body weight, CL: corpus length, CC: chest circumference, TL: thigh length, TC: thigh circumference, SC: shank circumference, SL: shank length: KL: keel length:, and BL: body length.

Figure 1 depicts correlation coefficients for predicting the correlation between body weight and the chickens' measured linear body measurements. The body weight of the chickens was positively and highly correlated ( p<0.01 ) with CC (0.74), TC (0.73), KL (0.73), CL (0.71), and BL (0.61). However, it was moderately and positively correlated ( p<0.05 ) with shank length (SL) (0.50), SC (0.46), and TL (0.43). On the other hand, the KL showed a positive highly significant correlation ( p<0.01 ) with CL ( r=0.67 ).

**Table 2:** MARS model.

Variables	Coeffıcıents
Intercept	1.85
h(CC-30.1)	0.06
h(TC-10.9)	0.38
h(TC-11.6)	-0.37
h(SL-13)	0.15
h(13.9-KL)	-0.11
h(KL-13.9)	0.39

CC: chest circumference, TC: thigh circumference, SL: shank length, and KL: keel length.

Table 2 below shows the findings of the MARS data-mining algorithm model. The MARS model created showed that CC, TC, SL, and KL were included in the established model. The model's first term had an intercept with a coefficient of 1.85. The second term, CC, had a cut-point of 30.1 cm for a positive coefficient of 0.60. The third term, TC, had a cut-point of 10.9 cm, with a positive coefficient of 0.38. The fourth term, TC, had a cut-point of 11.6 cm, with a negative coefficient of 0.37. The fifth term, SL, had a cut-point of 13 cm, with a positive coefficient of 0.15.

Meanwhile, the sixth term, TC, had a cut-point of 13.9 cm, with a negative coefficient of -0.11 . The last term was KL, with a positive coefficient of 0.39. The basic functions that reduce the model's performance obtained after the forward and backward pass stages were eliminated due to the GCV in MARS modeling.

**Figure 2:**
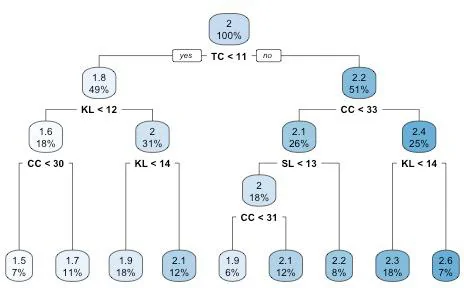
CART model predicting body weight of Kuroiler chickens from linear body measurements. TC: thigh circumference, KL: keel length, CC: chest circumference, and SL: shank length.

At the topmost node (node 0) of the regression tree diagram generated by CART (Fig. 2), the root node showed the overall body weight of chickens to be 2 kg. The node was split into two sub-groups based on TC, with a cut-off point of 11 cm. For the first sub-group, where TC was less than 11 cm, the average body weight was 1.8 kg, representing 49 % of the flock. When TC was greater than 11 cm, the obtained body weight was 2.2 kg, representing 51 % of the flock. The first sub-group of the tree was further split into two sub-groups based on KL, with a cut-point of 12 cm. When the KL was less than 12 cm, the mean BW was 1.6 kg, representing 18 % of the flock. When KL is greater than 12 cm, the mean BW is 2 kg, representing 31 % of the flock. This part of the tree was further divided into two sub-groups based on CC, with a cut-point of 33 cm. The results further revealed that, when the CC is less than 33 cm, the obtained BW is 2.1 kg, representing 26 % of the flock. When CC is greater than 33 cm, the obtained mean BW is 2.4 kg, representing 25 % of the flock. This part of the tree was divided into two sub-groups based on SL, with a cut-point of 13 cm. When the SL is less than 13 cm, the obtained BW was 2 kg, representing 18 % of the flock. When SL is greater than 13 cm, the obtained BW was 2.2 kg, representing 8 % of the flock.

The tree was further divided into two sub-groups for CC with a cut-point of 31 cm. This happened with the realization of TC >11 cm, CC <33 cm, SL <13 cm, and CC <31 cm with an obtained BW of 1.9 kg. In addition, in the case of TC >11 cm, CC <33 cm, SL < 13 cm, and CC >31 cm as the end nodes, the obtained BW was 2.1 kg. The tree was divided into two sub-groups for KL, with a cut-off point of 14 cm. This happened with the realization of TC >11 cm, CC > 33 cm, and LW <14 cm, with an obtained BW of 2.3 kg. In addition to the case of TC >11 cm, CC >33 cm, and LW > 14 cm as the end nodes, the obtained BW was 2.6 kg. The tree was divided into two sub-groups for CC with a cut-off point of 30 cm. This happened with the realization of TC <11 cm, KL < 12 cm, and CC <30 cm, with an obtained BW of 1.5 kg. In addition, in the case of TC <11 cm, LW <12 cm, and CC >30 cm as the end nodes, the obtained BW was 1.7 kg. The tree was divided into two sub-groups for KL, with a cut-point of 14 cm. This happened with the realization of TC <11 cm, KL >12 cm, and KL < 14 cm, with an obtained BW of 1.9 kg. In addition to the case of TC <11 cm, LW > 12 cm, and KL >14 cm as the last node, the obtained BW was 2.1 kg.

**Table 3:** Goodness-of-fit criteria for MARS and CART algorithms.

criterion	MARS		CART		Decision
	Training	Test	Training	Test	
Pearson's correlation coefficient^®^	0.90	0.91	0.91	0.87	Greater is better
Root-mean-square error (RMSE)	0.13	0.16	0.13	0.17	Smaller is better
Relative root-mean-square error (RRMSE)	6.54	8.11	6.23	8.33	Smaller is better
Standard deviation ratio (SDR)	0.43	0.46	0.41	0.49	Smaller is better
Coefficient of variation (CV)	6.59	7.99	6.27	8.47	Smaller is better
Mean absolute percentage error (MAPE)	5.57	6.64	4.98	6.60	Smaller is better
Relative approximation error (RAE)	0.00	0.01	0.00	0.01	Smaller is better
Akaike's information criterion (AIC)	-275.70	-87.29	-296.76	-99.77	Smaller is better
Performance index (PI)	3.44	4.25	3.23	4.45	Smaller is better
Mean error (ME)	0.00	-0.04	0.00	-0.01	The expected value is zero
Mean absolute deviation (MAD)	0.11	0.13	0.10	0.13	Smaller is better
Coefficient of determination (Rsq)	0.82	0.77	0.83	0.76	Greater is better
Adjusted coefficient of determination (Arsq)	0.80	0.69	0.83	0.76	Greater is better

### Comparison of the MARS and CART data-mining algorithms

With the goodness-of-fit criteria worked out for measuring the predictive performance, the training data set was used to make a decision between the MARS and CART data-mining algorithms, and the results are summarized in Table 3. The results revealed that CART had higher predictive performance in the criteria as compared to the MARS model. The CART data-mining algorithm showed smaller RRMSE, SDR, CV, MAPE, AIC, PI, and MAD test results. Nonetheless, the CART data-mining algorithm showed higher r and Arsq.

## 4 Discussion

Linear body measurement traits have been used in several studies to predict the body weight of livestock. Like this study, other studies revealed a significant correlation between body weight and linear body measurements. Specifically, this study's findings revealed a highly significant correlation between body weight and chest circumference, thigh circumference, keel length, corpus length, and body length, and a moderately significant correlation with shank length, shank circumference, and thigh length. These findings are comparable with those of Urooj et al. (2023), who reported a strong correlation between body weight, wing length, and chest circumference in Ross 308 broiler chickens. Furthermore, Habashy et al. (2021) also published findings akin to those in this study, where body weight was highly correlated with body length and moderately correlated with shank length at 12 weeks. The positive and strong correlations between traits mean that, if selection for any of these traits is made, it will result in the corresponding increase in the body weight of Kuroiler chickens. However, in contrast, Abdel-Lattif (2019) reported that chest circumference was not significantly correlated with body weight in both the Coshin and local chickens in Iraq. The correlations between the studied traits are suggestive of the fact that the traits are controlled by the same or related genes (Mathapo et al., 2022). Nonetheless, the discrepancies in the correlations and their strengths between different studies could be attributed to different species subjected to different management styles in different studies.

The correlation only informs us of the magnitude and direction but does not inform us of how much the independent variables contribute to the dependent variable. The current study employed the multivariate adaptive and regression spline (MARS) and the classification and regression tree (CART) models. The MARS model revealed that chest circumference, thigh circumference, shank length, and keel length were the determinant factors for predicting the body weight of Kuroiler chickens. The findings indicated that keel length was the best determinant factor, followed by chest circumference. A similar study by Tyasi et al. (2020a) reported wing length to be the best determinant factor of body weight of Hy-Line Silver Brown chicken. Despite the limited literature reporting the use of MARS in predicting body weight from linear body measurements in chickens, MARS has been used successfully in other species to predict body weight from linear body measurements. For instance, Faraz et al. (2023) reported on models that could predict the body weight of the Kajli sheep breed. Similarly, Hlokoe et al. (2022) also reported on models generated by MARS to predict the body weight of Nguni cows from linear measurements.

CART findings revealed that thigh circumference, keel length, chest circumference, and shank length were critical determinant factors for predicting the body weight of Kuroiler chickens. The current study's findings further revealed that thigh circumference was the best determinant factor for predicting body weight. The findings of Tyasi et al. (2020b) were in contrast with the current study's findings, where wing length was the best determinant trait for predicting body weight. Similar results were reported by Tyasi et al. (2021), who found that wing length was a contributing factor to the prediction of body weight in chickens. Dzungwe et al. (2018) and Chimezie et al. (2013) reported similar results, with wing length being used to predict body weight in chickens using traditional models. Oyebanjo et al. (2023) and Mallam et al. (2023) reported that the CART model revealed heart girth to be the best predictor of body weight in Red Sokoto and Sahel goats and Nigerian non-descript indigenous goats, respectively.

A comparison of the two models revealed that both MARS and CART identified keel length, shank length, chest circumference, and thigh circumference as significant predictors of the body weight of Kuroiler chickens. A goodness-of-fit test was then conducted to determine which of the two data-mining algorithms had a better predictive performance. The results indicated that the classification and regression tree (CART) model outperformed the multivariate adaptive regression spline (MARS) model. Assan et al. (2024) also reported that CART performed better than MARS in predicting the body weight of three (3) indigenous chickens. Despite the limited literature on the performance of the two algorithms in predicting body weight in chickens, CART and MARS have been compared comprehensively in other livestock species. Çelik et al. (2017) reported that the CART model performed well in predicting the body weight of Mengali rams of Pakistan. Hlokoe et al. (2022) reported that MARS performed better than CART in the prediction of the body weight of Nguni cows. However, Bila et al. (2023) reported that CART performed better than MARS in predicting the body weight of South African Sussex cattle. Urooj et al. (2023) reported that the ensemble model outperformed the MARS and CART models in broiler chickens. These reports, taken together with this study, indicate that the performance of these data-mining algorithms varies with species.

## 5 Conclusions

In the quest to find the most suitable model to predict body weight from linear body measurement traits, this study compared the predictive performance between MARS and CART data-mining algorithms. This study established that CART had a better predictive performance than MARS. However, chest circumference, thigh circumference, shank length, and wing length were isolated as significant predictors of the body weight of Kuroiler chickens by both MARS and CART. The CART model concludes that TC alone was the best explanatory variable for the prediction of the body weight of Kuroiler chickens in the absence of weighing scales. These can, therefore, be used not only to predict body weight but also to select for increased body weight as they also registered highly significant correlations with body weight.

## Data Availability

The data used in this study are available on request from the corresponding author.
